# Risk and Adverse Outcome Factors of Severe Acute Malnutrition in Children: A Hospital-Based Study in Odisha

**DOI:** 10.7759/cureus.18364

**Published:** 2021-09-28

**Authors:** Kedarnath Das, Swarnalata Das, Suchismita Mohapatra, Arakhita Swain, Nirmal K Mohakud

**Affiliations:** 1 Paediatrics, Srirama Chandra Bhanja Medical College and Hospital, Cuttack, IND; 2 Paediatrics, Kalinga Institute of Medical Sciences, Bhubaneswar, IND

**Keywords:** nrc, f-75 and f-100, complimentary feeding, socio-economic status, sam, malnutrition

## Abstract

Background

Malnutrition is prevalent in 41% of children less than five years old in developing countries.

Objective

To determine the clinical spectrum, identify the risk factors, and find out the factors responsible for the adverse outcomes of severe acute malnutrition (SAM) in children.

Methods

In this prospective cohort, children aged one month to five years with SAM from October 2016 to September 2018 were enrolled. Clinical profile, contributing factors, treatment, and outcome of cases (n=198) were noted.

Results

SAM was diagnosed in 323 (1.6%) of admitted cases. The unimmunized children were 123 (62.1%). Common co-morbidities were acute gastroenteritis (n=89, 44.9%), respiratory tract infection (n=88, 44.4%), and septicemia (n=54, 26.7%). Children not on exclusive breastfeeding (n=157, 79.1%), early complementary feeding (<6 months) (n=157, 88.2%), bottle-feeding (n=138, 77.55%), low birth weight (157, 79.1%), living in kutcha houses (115, 58.2%), and unavailability of safe drinking water (131, 66.4%) were the significant risk factors. Pneumonia, diarrhea, nutritional edema, hypothermia, and circulatory shock at the time of admission were responsible for adverse outcomes. One hundred and eighty-three (92.4%) children were cured and discharged and 15 (7.6%) children died.

Conclusions

Wrong feeding practices and unavailability of safe drinking water have an important bearing on the development of SAM children. Pneumonia, diarrhea, nutritional edema, hypothermia, and circulatory shock at the time of admission were responsible for adverse outcomes.

## Introduction

Malnutrition attributes to 33% of global deaths and 45% of deaths in under-five children in South Asia and Sub-Saharan Africa [1,2]. In India, nearly 57 million children are moderate to severely malnourished and account for more than 50% of deaths in the 0-4 years age group. Moreover, 48% of under-five children are stunted due to severe malnutrition [3]. Severe acute malnutrition (SAM) is managed as per the 10 steps of standardized inpatient treatment as per the 2016 modification of guidelines [4]. The ready-to-use therapeutic foods F-75 and F-100 have increased the feasibility of community management. Despite this standardized protocol of care, in-patient mortality is as high as 10%-40% [4], and the reasons are yet to be elucidated. It might be due to various co-morbidities associated with SAM children, improper adherence to the treatment protocol, defective management, and other socio-demographic causes. In India, more than 33% of deaths under five years of age are associated with malnutrition [3]. This might be due to change in innate and adaptive immunity as a result of nutrient and micronutrient deficiencies [5]. Besides these, associated co-morbidities like anemia, diarrhea, dehydration, hypoglycemia, hypothermia, electrolyte imbalance, and sepsis play a major role in increased mortality [2].

Even though the prevalence of malnutrition is very high and is a leading cause of under-five mortality, little data are available regarding its clinical spectrum and determinants of SAM in Odisha, and the eastern part of India [6]. This study aims at determining the clinical spectrum, predictors, socio-demographic factors, and outcomes associated with SAM. Besides, the outcome of the study will help in improvisation in the management protocol of such children in this part of the country.

## Materials and methods

**Figure 1:**
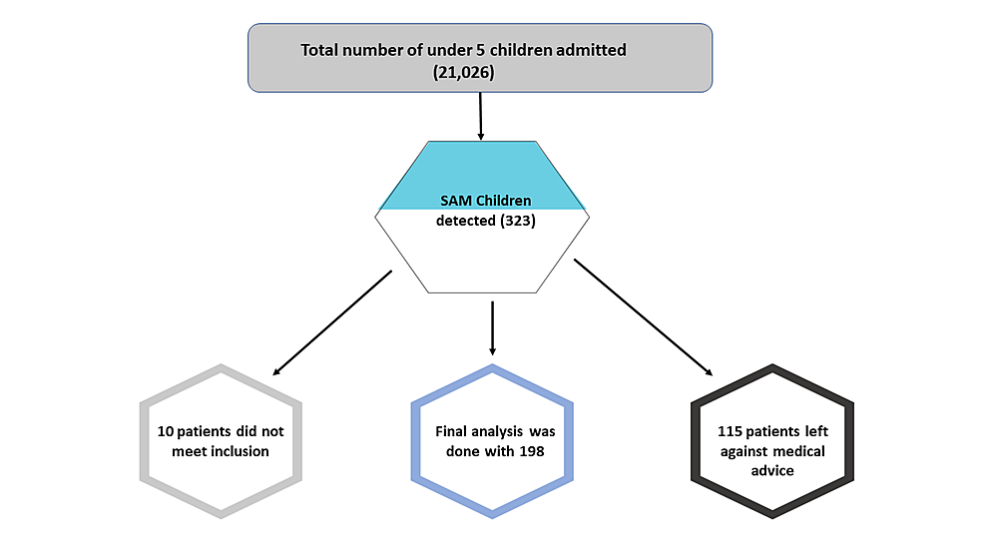
Flowchart of enrollment of study participants SAM, severe acute malnutrition

This was a prospective observational study done during the period from October 2016 to September 2018 in children aged one month to five years, admitted in the pediatric ward at SCB Medical College and SVPPGIP, Cuttack, in the Central part of Odisha. Children diagnosed to have SAM as per World Health Organization (WHO) criteria were included (Figure 1).

All children diagnosed with SAM were admitted to the pediatrics ward or nutritional rehabilitation center. Approval of the institutional ethical committee (Sriram Chandra Bhanja Medical College, Cuttack, Odisha) (IEC/IRB No:661/2018) was obtained for the study.

SAM for 0-5 months of age is defined as weight-for-length <-3 z-score of the WHO child growth standard median or presence of bilateral pitting edema. For children six months to five years, weight-for-length < -3 z-score of the WHO child growth standard median and mid-upper-arm circumference (MUAC) <115 mm or presence of bilateral pitting edema [7]. Children with secondary malnutrition (malabsorption syndromes, cerebral palsy, congenital heart disease, metabolic disease, immunodeficiency disorder, chronic kidney disease, chronic lung disease, HIV), leaving the hospital before the completion of treatment, and non-nutritional causes of edema like nephrotic syndrome were excluded. Demographic details like age, sex, rural/urban, socioeconomic status, and clinical information were collected.

For children <2 years of age, weight and length were measured by using a Salter hanging scale and length board, while for children between two and five years of age weight and length were measured by a weighing machine (Virgo, fully automated, 0-100 kg, accuracy: ±10 g) and a stadiometer. Weight for height/length and z score of less than -1 was indicated as mild, -2 was indicated as moderate, and -3 was indicated as severe wasting. The MUAC was taken on the left arm in the midpoint between the olecranon process and acromion.

Anemia is defined in <6 months as hemoglobin (Hb) <12 g/dL and 6 months-5 years as Hb <11 g/dL. Hypoglycemia is defined as a blood glucose level <54 mg/dL. Laboratory investigations were done for diagnosis as well as the exclusion of common illness and associated co-morbidities. All the SAM children were managed with updated WHO guidelines [8].

The discharge criteria included weight gain of at least 15%; resolved pedal edema (if present); and achievement of -1 SD or -2 SD on weight-for-height chart, according to age and gender.

Data analysis

Descriptive statistics were calculated using SPSS software version 21 (IBM Corp, Armonk, NY). Data were summarized in frequency tables. Variables were compared using logistic regression analysis. A P-value less than 0.05 was considered statistically significant.

Predictor variables

Age, sex, socio-economic status, immunization status, feeding practices, perinatal history, socio-demographic factors, household, and environmental history were used as predictors of SAM, whereas the presence of fever, diarrhea, vomiting, weight loss, edema, dehydration, visible severe wasting, cough and cold, anemia, edema, shock, hypoglycemia, hypothermia, sepsis, and electrolyte imbalance was considered as predictors of outcome of SAM.

Outcome variable

Outcomes of malnutrition were recovery or death.

## Results

Table 1 showed that majority of SAM children (n=129, 65.5%) were between 6 and 24 months. There was a male (108, 54.6%) predominance. Large number of children (169, 85.3%) were from rural background. Almost all SAM children (191, 96.5%) belong to lower socio-economic group. The non-immunized children were 123 (62.1%). Only 27 (13.7%) children had completed immunization. They mostly presented with fever (n=140, 70.6%), cough and cold (n=94, 47.6%), diarrhea (n=112, 56.8%), weight loss (n=98, 49.7%), and lethargy (n=100, 50.8%). 

**Table 1:** Demographic profile and clinical spectrum of severe acute malnutrition in children

Parameters	Frequency (%)
Age	
<6 months	13 (6.5)
6-24 months	129 (65.5)
>24-59 months	56 (28.4)
Male	108 (54.6)
Rural	169 (85.3)
Urban	29 (14.7)
Socioeconomic status	
Lower	40 (20.2)
Upper lower	123 (61.8)
Lower middle	28 (14)
Upper middle	5 (2.5)
Upper	2 (1)
Immunization status as per age	
Completed	27 (13.7)
Incomplete	48 (24.2)
Non-immunized	123 (62.1)
Fever	140 (70.6)
Diarrhea	112 (56.8)
Vomiting	101 (51)
Cough and cold	94 (47.6)
Weight loss	98 (49.7)
Edema	57 (28.7)
Lethargy	100 (50.8)
Dehydration	30 (15.4)
Visible severe wasting	66 (33.4)
Skin changes	70 (35.6)
Hair changes	49 (24.6)
Signs of vitamin deficiency	55 (27.7)

Social risk factors associated with SAM children are shown in Table 2. Children not on exclusive breastfeeding (n=157, 79.1%), starting of complementary feeding in <6 months of age (n=157, 88.2%), bottle-feeding infants (n=138, 77.55%), low-birth-weight babies (157, 79.1%), children living in kutcha houses (115, 58.2%), and unavailability of safe drinking water (131, 66.4%) were risk factors for development of SAM in children (P<0.05). On univariate analysis, all the above factors had a significant odds ratio. Maternal age between 21 and 30 years constitutes the major group (n=112, 58.5) at the time of childbirth. The number of young mothers (<20 years) was 69 (34.8) and statistically nonsignificant for the adverse outcome (P>0.05). 

**Table 2:** Social risk factors in SAM children (n=198) SAM, severe acute malnutrition; Ref, references; OR, odds ratio

Parameters		Number of cases (%)	OR	P-value
Maternal age at the time of childbirth	<20 years	69 (34.8)	1.213	>0.05
	21-30 years	116 (58.5)	Ref	
	>30 years	13 (6.7)	0.067	>0.05
Prelacteal feed	Given	117 (59.1)	1.326	>0.05
	Not given	81 (40.9)	Ref	
Duration of exclusive breastfeeding	<6 months	157 (79.1)	4.676	<0.05
	$\geq$6 months	41 (20.9)	Ref	
Colostrum	Given	86 (43.4)	0.127	>0.05
	Not given	112 (56.6)	Ref	
Age of starting complementary feeding (n=178)	<6 months	157 (88.2)	6.367	<0.05
	6-12 months	17 (9.5)	Ref	
	>12 months	4 (2.3)	3.528	>0.05
Method of feeding (n=178)	Katori spoon	34 (19.1)	Ref	
	Bottle feeding	138 (77.5)	4.325	<0.05
	Both katori and bottle	6 (3.4)	0.873	>0.05
Birth weight of the child	<2.5 kg	157 (79.1)	3.487	<0.05
	$\geq$2.5 kg	41 (20.9)	Ref	
Type of house	Kutcha	115 (58.2)	1.367	<0.05
	Pucca	83 (41.8)	Ref	
Use of sanitary latrine	Yes	52 (26)	Ref	
	No	146 (74)	3.474	<0.05
Safe drinking water supply	Present	67 (33.6)	Ref	
	Absent	131 (66.4)	2.563	<0.05

The most common co-morbidities associated with malnutrition were acute gastroenteritis (n=89, 44.9%), acute respiratory tract infection (ARI) (n=88, 44.4%), septicemia (n=54, 26.7%), and urinary tract infection (UTI) (n=51, 25.7%). Tuberculosis and measles were found in 6 (3%) cases each (Table 3). 

**Table 3:** Associated co-morbidities and complications in SAM children SAM, severe acute malnutrition; ARI, acute respiratory tract infection; TB, tuberculosis; UTI, urinary tract infection

Co-morbidities	Number of cases	%
Acute gastroenteritis	89	44.9
ARI	88	44.4
Sepsis	54	26.7
UTI	51	25.7
Malaria	9	4.5
Measles	6	3
TB	6	3
Otitis media	5	2.5
Complications		
Anemia	96	48.5
Severe dehydration	62	31.5
Sepsis	54	27.4
Hypothermia	36	18.4
Hypoglycemia	29	14.7
Electrolyte imbalance	8	4.2
Shock	7	3.7
Outcome		
Recovered	183	92.4
Death	15	7.6

Table 4 depicts various predictors associated with the outcome of SAM children. Factors like dietary risk factors (107, 58.1%), edematous malnutrition (38, 20.5%), lethargy, pneumonia, hypothermia, and shock at the time of admission were associated with adverse outcomes. 

**Table 4:** Various predictors associated with adverse outcomes of SAM children SAM, severe acute malnutrition Dietary risk factors include prelacteal feed, early starting complementary feeding, top feeding, and bottle feeding

Variables		Outcome		P-value
		Cured (n=183)	Death (n=15)	
Socioeconomic status	Upper	5 (2.6%)	0 (0.0%)	>0.05
	Upper middle	52 (28.2%)	1 (7.7%)	
	Lower middle	73 (40.2%)	8 (53.8%)	
	Upper lower	50 (27.4%)	6 (38.5%)	
	Lower	3 (1.7%)	0 (0.0%)	
Complete immunization	Yes	147 (80.3%)	12 (76.9%)	>0.05
	No	36 (19.7%)	3 (23.1%)	
Dietary risk factors	Present	107 (58.1%)	12 (76.9%)	<0.05
	Absent	76 (41.9%)	3 (23.1%)	
Perinatal risk factors	Present	30 (16.2%)	8 (53.8%)	>0.05
	Absent	153 (83.8%)	7 (46.2%)	
Socio-demographic risk factors	Present	3 (1.7%)	9 (61.5%)	>0.05
	Absent	180 (98.3%)	6 (38.5%)	
Housing and environmental risk factors	Present	63 (34.2%)	13 (84.6%)	>0.05
	Absent	120 (65.8%)	2 (15.4%)	
Diarrhea	Present	61 (33.3%)	7 (46.2%)	<0.05
	Absent	122 (66.7%)	8 (53.8%)	
Cough and cold	Present	50 (27.4%)	9 (61.5%)	<0.05
	Absent	133 (72.6%)	6 (38.5%)	
Edema	Present	38 (20.5%)	7 (46.2%)	<0.05
	Absent	145 (79.5%)	8 (53.8%)	
Lethargy	Present	83 (45.3%)	15 (100%)	<0.05
	Absent	100 (54.7%)	0 (0.0%)	
Dehydration	Present	20 (11.1%)	8 (53.8%)	<0.05
	Absent	163 (88.9%)	7 (46.2%)	
Visible severe wasting	Present	52 (28.2%)	7 (46.2%)	>0.05
	Absent	131 (71.8%)	8 (53.8%)	
Anemia	Present	89 (48.7%)	7 (46.2%)	>0.05
	Absent	94 (51.3%)	8 (53.8%)	
Hypothermia	Present	50 (27.4%)	9 (61.5%)	<0.05
	Absent	133 (72.6%)	6 (38.5%)	
Hypoglycemia	Present	17 (9.4%)	10 (69.2%)	>0.05
	Absent	166 (90.6%)	5 (30.8%)	
Sepsis	Present	3 (1.7%)	9 (61.5%)	>0.05
	Absent	180 (98.3%)	6 (38.5%)	
Electrolyte imbalance	Present	178 (97.4%)	14 (92.3%)	>0.05
	Absent	5 (2.6%)	1 (7.7%)	
Shock	Present	5 (2.6%)	13 (84.6%)	<0.05
	Absent	178 (97.4%)	2 (15.4%)	

The average length of hospital stay among SAM children was 19.53 + 9.54 days (range, 3-35 days). The length of hospital stay was more in the presence of shock (P<0.05), hypoglycemia (P<0.05), lethargy (P<0.05), and sepsis (P<0.05) at the time of admission. A total of 183 (92.4%) SAM children were cured and discharged, whereas 15 (7.6%) cases died. Septicemia contributes to more than one-fourth of death cases. 

## Discussion

This study was intended to identify the comorbidities, treatment outcomes, and various predictors associated with the outcome of SAM children. Prevalence of SAM is more common among children 6-24 months of age. It might be due to the initiation of poorly prepared complementary food leading to frequent gastrointestinal tract infections. Similar findings were reported in previous studies [9,10]. Interestingly infants under six months constituted 6.5% of cases. A study from Cameroon by Chiabi et al. (2017) explored malnutrition in children <6 months of age, consistent with our finding [9]. This is due to prelacteals, faulty feeding practices, early weaning, and bottle feeding being detected in the study. Though colostrum is a very important food to start with, 112 (56.6%) newborn babies had not received it in our study. However, it is not associated with the risk factor for the development of SAM in later life (P>0.05). But deprivation of colostrums and receiving prelacteals at birth have been found to have increased risk of malnutrition in other studies [11,12].

Co-morbidities like acute gastroenteritis, ARI, sepsis, UTI, and anemia lead to poor nutritional recovery. We have identified seven statistically significant risk factors associated with increased mortality: dietary risk factors, pneumonia, diarrhea, dehydration, nutritional edema, hypothermia, and circulatory shock. Derseh et al. reported a similar presentation in Ethiopia [13]. These factors along with comorbidities contribute toward a decompensation of physiological pathways and impaired immune system leading to the severity of SAM. Interventions like proper immunization, sanitation, safe drinking water, and universal health coverage are potential ones for better outcomes [14].

The occurrence of recurrent or chronic diarrhea leads to malnutrition [15]. This is due to a decrease in appetite and malabsorption and depressed immunity and a vicious cycle of diarrhea. These episodes are an economic burden to the families in a developing country [16]. Introduction of prelacteals, not on exclusive breastfeeding for six months, and introduction of complementary food before six months had a significant association with SAM as was found in this study. Previous studies had similar reports [10,17]. Association of infections like ARI (pneumonia), sepsis, and UTI reduces the physiological reserve of SAM children and increases metabolic demand to contain fever, work of breathing, and cardiac output. These children become hypoxic and have a further decrease in appetite. All these lead to decreased food and calories intake and many a time they may need nasogastric tube feeding, resulting in malnutrition.

Around half of the SAM children are accompanied by anemia (n=89, 48.7%) due to increased demands for iron in this growing phase of life. Iron deficiency is due to inadequate iron in the diet, micronutrient deficiencies, and infections like malaria, measles, tuberculosis, and hookworm infestation. Iron deficiency leads to impaired innate and adaptive immunity and is thereby vulnerable to infections [18,19].

The SAM children presenting with shock representing a compromised physiological state and end-organ dysfunctions were more likely to have increased risk of death among the admitted cases. These groups of children have poor physiological reserve, and while resuscitating there was more chance of fluid overload leading to poor outcome.

This study found that younger maternal age (<20 years) did not influence the occurrence of SAM. But other studies had reported that maternal age below 25 years is a risk factor for severe malnutrition [20,21]. In this study, low birth weight (<2.5 kg) was an independent risk factor for SAM. A similar conclusion was drawn by the Mukuku et al. (2019) study [20]. The low birth weight of babies is due to malnutrition of mothers, which indicates poor socio-economic condition, defective feeding, and environmental hygiene practices in the family. So a child who is already malnourished prior to birth and continues to live in the same condition will suffer from malnutrition that persists or worsens.

According to WHO, a death rate of SAM children <10% is acceptable and >15% is alarming [22]. The mortality of cases in this study was 7.6%, which is within the global range indicating the benefits of using an updated guideline for inpatients. As septicemia is the major contributor, aggressive treatment is needed in every suspected case with suitable antibiotics to prevent mortality and morbidity in SAM children.

Limitations

This hospital-based observational study could not focus on the true prevalence of SAM in the community. This small piece of study necessitates and stimulates a study comprising a larger sample for a longer duration to provide a true picture of the burden and outcome of SAM.

## Conclusions

SAM is prevalent in children of 6-24 months of age. Adherence to updated WHO guidelines for inpatient management has helped to reduce mortality to an acceptable level. Dietary risk factors such as duration of exclusive breastfeeding, bottle feeding, and delayed introduction of complementary feeding were significantly associated with poor outcomes. Screening should be routinely performed in all healthcare centers in the community for early detection of SAM children. Immunization and universal health coverage to all will help to reduce malnutrition and mortality among SAM children.
